# Shaping healthcare-seeking processes during fatal illness in resource-poor settings. A study in Lao PDR

**DOI:** 10.1186/1472-6963-12-477

**Published:** 2012-12-22

**Authors:** Helle M Alvesson, Magnus Lindelow, Bouasavanh Khanthaphat, Lucie Laflamme

**Affiliations:** 1 Department of Public Health Sciences, Division of Global Health, Karolinska Institutet, Nobels väg 9, Stockholm, 171 77, Sweden; 2 Human Development Department, The World Bank, Brazil SCN, Quadra 2, Lote A. Ed. Corporate Center, 7th andar, Brasilia, DF, 70712-900, Brazil; 3 Indochina Research Laos Ltd, IRL Building, 282/17 Phontong-Savath, PO Box 1887, Vientiane Capital, Chanthabouly District, Laos

**Keywords:** Healthcare-seeking behavior, Maternal and child mortality, Health systems, Qualitative interviews, Lao PDR

## Abstract

**Background:**

There are profound social meanings attached to bearing children that affect the experience of losing a child, which is akin to the loss of a mother in the household. The objective of this study is to comprehend the broader processes that shape household healthcare-seeking during fatal illness episodes or reproductive health emergencies in resource-poor communities.

**Methods:**

The study was conducted in six purposively selected poor, rural communities in Lao PDR, located in two districts that represent communities with different access to health facilities and contain diverse ethnic groups. Households having experienced fatal cases were first identified in focus group discussions with community members, which lead to the identification of 26 deaths in eleven households through caregiver and spouse interviews. The interviews used an open-ended anthropological approach and followed a three-delay framework. Interpretive description was used in the data analysis.

**Results:**

The healthcare-seeking behavior reported by caregivers revealed a broad range of providers, reflecting the mix of public, private, informal and traditional health services in Lao PDR. Most caregivers had experienced multiple constraints in healthcare-seeking prior to death. Decisions regarding care-seeking were characterized as social rather than individual actions. They were constrained by medical costs, low expectations of recovery and worries about normative expectations from healthcare workers on how patients and caregivers should behave at health facilities to qualify for treatment. Caregivers raised the difficulties in determining the severity of the state of the child/mother. Delays in reaching care related to lack of physical access and to risks associated with taking a sick family member out of the local community. Delays in receiving care were affected by the perceived low quality of care provided at the health facilities.

**Conclusions:**

Care-seeking is influenced by family- and community-based relations, which are integrated parts of people’s everyday life. The medical and normative responses from health providers affect the behavior of care-seekers. An anthropological approach to capture the experience of caregivers in relation to deciding, seeking and reaching care reveals the complexity and socio-cultural context surrounding maternal and child mortality and has implications for how future mortality data should be developed and interpreted.

## Background

Recent estimates suggest that 8.1 million children under-5 years of age die annually [1], with 40 percent of those deaths occurring in the neonatal period [2]. At the same time, around 350 000 women die in pregnancy, childbirth or soon after [2]. Those mother and child deaths affect some countries to a far greater extent than others and much remains to be understood about the processes prevailing prior to their occurrence for effective preventative or curative measures to be put into place [3]. There are profound social meanings attached to bearing children that affect the experience of losing a child [4,5], which is akin to the loss of a mother in the household [6]. These meanings are shaped by expectations and relationships within the family and the community, including but not restricted to those in relation to health care providers [7-9]. Did caregivers or spouses recognize the need for seeking care and did they actually seek care? Did mothers and children receive appropriate health care services at the right time? If not, what were the constraints faced?

The design of contextualized studies of why loss in childbearing is still a serious public health problem has recently been encouraged by “joining-up thinking” from multi-disciplinary perspectives [10]. The “thinking” highlights the importance of non-medical factors that may play a determinant role in understanding healthcare-seeking behaviors such as the gender aspects of decision-making and local perceptions of illness and treatment [11-16]. The local and contextual processes of how loss and the risk of loss are interpreted provide insights, not only on the sequencing of seeking treatment, but also on caregivers’ rationales for preferences for private and public care.

Access to data on maternal and child mortality is crucial to answering questions related to constraints in seeking or receiving care. Traditional mortality data are based on vital registers and do not help answering those questions. For that reason, and also because mortality data are missing in contexts where they are most pressingly needed, the manner in which mortality data are gathered has been revisited [17-22]. Social, behavioral and health system determinants of maternal and child mortality are recognized as increasingly important for understanding why children and mothers die [21]. In building up national health statistics, the point has been made that the country’s needs and capacities have to be prioritized, at the same time as the global demand for monitoring the Millennium Development Goals is also important to satisfy [17].

Against this background, this study aims to cast light on the broader processes that shape household healthcare-seeking during fatal illness episodes or reproductive health emergencies. Focusing on poor communities in Lao PDR, one of the countries most affected by maternal and child mortality, we reconstruct healthcare-seeking processes preceding fatal illness, building on the narratives of caregivers gathered during open-ended individual interviews [23]. Those narrative interviews closely follow the anthropological tradition where people’s life experience is at the center [24]. The qualitative in-depth interview can provide a situation of concentration and anonymity, which offers an occasion to retell the illness and put the episode into perspective for caregivers. The interview creates opportunities for the interviewer and the informant to not only reconstruct a process but also reflect upon it and, in light of this, evaluate events, situations and responses [25], which in turn is highly relevant in understanding the context of high mortality. There is reason to believe that the open-ended in-depth interview can set the stage for understanding those processes that frame maternal and child mortality [21,26].

## Methods

### Setting

In Lao PDR, maternal and child mortality remains high in spite of a steady decline in recent decades [1]. Only 20 percent of births are delivered by skilled birth attendants [27] and a worrying decline has been observed in the proportion of mothers seeking care for children with suspected pneumonia from 36 to 32 percent between 2000 and 2006 [28,29].

Lao PDR is organized in 16 provinces and one municipality; each province is divided into districts with a total of 139 districts (average number is eight districts per province) [30]. The formal health care system is organized in four administrative levels – central, province, district and village. There is one provincial hospital in each province and most districts have a district hospital, which in turn is assisted by health centers at village level. The private health care sector expanded in the 1980s as part of a major health financing reform, which in turn was a consequence of a shift from a planned to a market-based economy in Laos [31]. The expansion was also partly due to a lack of drug supply in the public sector. The Ministry of Health is currently trying to license unlicensed pharmacies and drug vendors [32]. The total number of unlicensed drug vendors or pharmacies is unknown and they are found in towns and also in remote areas with limited access to public care. Traditional practitioners (fortune-tellers, shamans and herbalists) are another popular source of care [33] and traditional (herbal) medicine is a high priority of the Lao Government. Traditional medicine is an integrated part of the Lao National Drug Policy [32].

Lao PDR is one of the world’s most linguistically and ethnically diverse countries with 49 officially recognized ethnic groups by the Lao government [30], which in turn from an anthropological perspective can be divided into around 230 ethno-linguistic groups [34]. The groups belong to one of four main linguistic families Lao-Tai, Mon-Khemer, Hmong-lu Mien, Chine-Tibet. The ethnic Lao-Tai generally practice Buddhism while the majority of the non-Lao ethnic groups adhere to traditional religious practices of Animism - even though there are variations in the local expressions and many of the religious practices show some degree of syncretism. The ethnic groups are also diversified in terms of livelihood and this ranges from hunting and gathering, different forms of swidden cultivation in the upland areas, to wet-rice farming in the lowlands.

Laos is one of the most inequitable countries in terms of women’s access to health care services such as skilled birth attendants and to antenatal care visits [35]. Women from rural areas also have much lower access than women in urban ones, which is partly explained by geographical constraints and higher poverty levels in rural areas [30]. Furthermore health facilities are dependent on revolving drug funds for revenue generation [32], which is more difficult to sustain in rural areas with low cash flows. Another factor is that the majority of health staff members are from the main ethnic group (Lao Loum), which means that when posted in remote rural areas they are far from their home. This can result in reduced opening hours at the health centres. The lack of a common language between health workers and non-Lao speaking users is also a constraint.

### Selection of participants

The data collection on which this article is based forms part of a broader study, which looked both at patient and provider perspectives on healthcare-seeking behavior and quality of care in public and private health outlets in Lao PDR. In the main study, data was collected through interviews with formal and informal health care providers as well as group discussions with men, women, pregnant women and grandmothers. The fieldwork took place in February and March 2009. For the main study we first selected two different provinces, and in each of them one district, aiming at geographic and ethnic diversity as well as access to care. The two provinces selected, Xekong and Savannakhet, are situated in the southern and central part of the country. They are among the poorest in the country with around 40 percent of the population living in poverty [36].

The districts were selected on the grounds that they had a functioning district hospital and some provision of private treatment from drug vendors or private doctors and nurses.^a^ These selection criteria reflect our objective of understanding caregiver preferences when they in fact had healthcare alternatives to choose between. Within each district, the data collection took place in six purposely selected rural communities of which four had “good access” (less than two hours walk) to a health center and two had “low access” (more than four hours walk). The purpose of having more communities with relatively good access to a health facility was to identify parents who had experienced a loss of a child within a local area in which healthcare providers in fact were available.

The two study districts have a total population of 55000 inhabitants; there is one district hospital in each of the districts and 5 respective 6 health centers in each district. These districts were predominantly rural, including both lowland farming areas and upland communities that tend to be poorer [30]. The study population is ethnically diverse, including different Buddhist Lao-Loum groups (around 65–75 percent of the population), in addition to many small minority groups of Tarieng, Alak and Mangkhong communities, which reflects the ethnic mosaic fairly well. However, the districts are ethnically tremendously diverse and the sample does not cover all the groups living in the districts.

In a third step, the District Health Office provided assistance in selecting the communities, which assured that our information on the travel time from community to health center was correct; that the health centers in the areas in fact were staffed and that different ethnic groups were represented in the sample. We aimed at selecting five families in each community based on consultation with the village authorities and health centers, as well as through one focus group discussion with women and one with men in each of the six communities. Through these various sources we gained knowledge on the fatal illness episodes in each village. The selection criterion was households experiencing a fatal health episode of a child under five years of age within the last 24 months [37]. The number of fatal health episodes of children under five was fewer than anticipated and we relaxed our inclusion criteria by adding non-fatal outcomes of severe illness. Households who had lost a newborn in the home during delivery or during the first or second day without consulting any providers were excluded from the sample, which implies an underreporting of delays in deciding and reaching a health facility. In a total of 11 households one or more fatal health episodes were identified. These 11 households reported 26 deaths (2 mothers and 24 children) and they are the focus of analysis (Table 1).

**Table 1:** Description of 26 fatal health episodes

**ID**	**Age**	**Sex**	**Wealth status**	**Ethnic group**	**Access to facility**	**Place and timing of death**	**Type of delay**
1	2 years	Boy	Middle	Lavee	Good	Home on 3rd day	1
2	1 year	Boy	Middle	Lavee	Good	Hospital after 20 months of ill health	3
3	5 months	Girl	Poor	Alak	Low	Home after 5 months of ill health	1, 3
4	12 years	Boy	Poor	Alak	Low	Home on 15th day of illness	1, 2
5	1, 2 and 3 days old	Triplets	Middle	Tarieng	Good	Home on 1st , 2nd and 3rd day after birth	1, 2, 3
6	Mother and fetus	Mother	Middle	Mangkhong	Good	Hospital 13 hours after labor started	3
7	3 days		Middle	Mangkhong	Good	Hospital on 3rd day after birth	1, 2,3
8	9 years	Girl	Middle	Lao Loum	Good	On way home from HC after 4 days of illness	
9	3years	Boy	Poor	Mangkhong	Low	On way home from the hospital on the 9th day of illness	1, 2
10	6 years	Girl	Poor	Mangkhong	Low	Home on 7th day	1
11	4 years	Boy	Poor	Mangkhong	Low	Home after 10 days of illness	1, 2
12	2 months	Twin girls	Middle	Lao Loum	Good	Home on 3rd and 4th day of illness	1, 2
13	1 month	Triplet boys	Middle	Lao Loum	Good	Home after 4 weeks of life	1, 3
14	3years	Girl	Poor	Lavee	Low	Home on 5th day	1,2
15	Stillborn		Poor	Alak	Good	Home	2
16	4 days	Girl	Poor	Lavee	Low	Home on 4th day of illness	1, 2
17	2 years	Girl	Poor	Mangkhong	Good	Home on 9th day of illness	1, 2, 3
18	2 years	Boy	Middle	Lavee	Good	At home on 6th day of illness	1, 3
19	Stillborn		Poor	Alak	Good	Home	
20	Woman	Mother	Poor	Mangkhong	Good	On way to hospital after delivery	1,2,3

Type of delay:

1 = delay in making a decision to seek care;

2 = delay in reaching the health facility;

3 = delay in receiving care at a health facility upon arrival.

### Interview process and framework

To reconstruct the healthcare seeking process surrounding each individual case, we used as a background the three-delay framework [38] so as to determine potential reasons for delays in seeking, reaching and receiving healthcare. This framework was originally developed for obstetric emergencies but it was also applied more recently to perinatal, newborn and under-5 death [26,39,40]. The in-depth interviews were semi-structured and the open-ended approach allowed for the coverage of a broad range of social, cultural and economic topics related to the circumstances of the deaths. The caregivers were encouraged to describe the episode from beginning to end in their own words, and were, during this illness narrative, prompted to describe decision-making processes, perceived causes, provider preferences, medical costs and perceived quality of care [23]. Follow-up questions assured that factors relevant for the three delays were captured. Basic information on the number of household members, language spoken and a self-rated socio-economic status (poor, middle, better-off) was also collected; the latter was complemented by our observations of roofing type and visible assets.

In each household the closest caregivers of the deceased were identified, which were the mother, both parents, or in one case a grandparent. At the beginning of some of the interviews, it became clear that some families had lost more children than the field team initially was aware of. These prior experiences had shaped some of the coping strategies in the more recent health shocks and have therefore been included in the analysis independently of the age of the child [41]. The interviews lasted around 1 ½ hours and were performed at the home of the caregivers in the Lao language by two Laotian moderators and note-takers with experiences in conducting qualitative interviews in rural communities in Lao. The interviews were recorded, supplemented by notes from note-takers and summarized into English.

### Data analysis

The interview summaries were extensively discussed by the field moderators and authors; during this process clarification and alternative interpretations of each interview were discussed. The illness narratives were analyzed with the delay-framework [38] by means of interpretive description [42,43]. We grouped factors influencing delay in seeking, reaching and receiving for each death. Within each delay, we looked for themes and patterns that could explain the social processes shaping the delays.

Delay in care-seeking was defined as patients not consulting any provider (including traditional and informal providers) within the first 24 hours after the caregivers recognized illness. The 24 hour limit was easy for caregivers to remember. We also collected information on self-medication, defined as when purchased drugs and drugs the household already had at home was used without the child had been seen by a provider. Factors related to the second delay of reaching care are based on caregivers’ description of the time it took to reach the facility and if there were problems on the way. In addition it includes perceived transportation delay, which means that caregivers did not look for means of transportation due to problems in organizing or paying for the transport or because they clearly expressed in the interview that they knew that the distance was too far to manage in the situation. The third delay, that of receiving treatment, is based on caregivers’ description of whether any services were received that made sense to the caregivers when considering the condition of their child or spouse.

The analysis is thus based on caregiver perceptions and has been done by non-medically trained researchers, which implies that we are not making medical statements about the quality or appropriateness of the care received at the health facilities. Rather, the notion of quality of care is based on the satisfaction level of caregivers and our purpose was to gain an understanding of their view of the consultations with health providers.

Ethical approval for the study was obtained from the National Ethic Committee on Health Research (N017/NECHR) in Lao PDR.

## Results

The health-seeking behaviors reported by the caregivers revealed a broad range of different providers, reflecting the mixed market of public, private, informal and traditional health services in Lao PDR [44]. The majority of patients (15 of 26) consulted a health worker prior to death (Table 2). Nine deceased had been seen by a health worker immediately prior to death. Self-medication with biomedicine purchased at authorized or unauthorized pharmacies including mobile drug vendors was reported as the only source of treatment in five of the deaths. In these cases, the patient was not brought to the drug vendor but a caregiver described the symptoms of the family member and antibiotics, malaria treatment or pain killers were purchased. Five patients had only been treated by traditional fortune-tellers, shamans or herbalists. Four of these five cases had poor access to biomedical treatment.

**Table 2:** Type of treatment prior to death

**Type of treatment prior to death**	**Number of deaths**
No treatment	1
Traditional medicine only	5
Self-treatment with biomedicine	5
Biomedical healthcare during last month	4
Biomedical healthcare during last week	2
Biomedical healthcare immediately prior to death	9
**Total**	26

Biomedical health care includes treatment by all types of providers trained by the Ministry of Health; Trained Birth Attendants, Village Health Volunteers, nurses and medical doctors.

Immediately prior to death is defined as patients who died at a health facility, in the presence of a Village Health Worker/nurse in the home, or died on the way home from a health facility a few hours after leaving.

Many of the episodes demonstrated that factors within the household, the community and the health system also influenced the utilization of health care services in areas with relatively good access to formal health facilities (Table 3). In all but two deaths at least one type of delay was identified.

**Table 3:** Number of deaths per type of delay

**Delay**	**Type of delay**	**Number of deaths**
Delay 1	Delay in making a decision to seek care	20
Delay 2	Delay in reaching the health facility	14
Delay 3	Delay in receiving care at a health facility upon arrival	14

The initial narrative of a mother who describes the days before the loss of her three year old son, illustrates the type of conversations that were held with caregivers and the way delays emerged:

"In the beginning, he had a fever but it was not a very high fever. I did not bring him anywhere - I thought the illness was not serious. I gave him paracetamol. One week later he got very sick. I and my husband brought him to the Health Center but there was no nurse there as it was Sunday. We had to take a “Tok Tok” [hand tractor] to the district hospital^*b*^. The doctor said that he had malaria and gave him a drip. He was crying and screaming because he was afraid of the needles……[he went into a cramp but came out of it again, inserted by author]……I asked the doctor to bring him home. The doctor said if I took him home he would die. At that time I thought his illness was caused by spirits and I would like to take him home and get him treated with a spiritual practitioner. The doctor warned me; he said that if I took him home he would die. But I insisted on bringing him back. He passed away on the way back to our house….….I and my husband are really saddened by this experience. It is because we have low education. We don’t know the cause of the disease so we always thought the illnesses were caused by spirits and therefore we wanted to bring our son back to be treated by a spiritual practitioner.….Until this day I am very upset about my decision. If we had not taken him out of the hospital he might not have died [ID 9]."

The interview continues with more probing questions on the symptoms, expectations, costs and causes. In our analysis, this case illustrates delay in decision-making of seeking care and delay in reaching care.

### Delay in care-seeking

In the caregiver narratives on the decision to seek or not seek care, the active selection of providers emerged as an important process. One of the main motives for seeking formal healthcare was to receive good quality of care, while the reasons for delaying or not seeking care were multi-faceted. First, disagreements within the household were identified as a very important cause of delay. In almost half of the households there had been some disagreement on type and timing of health-seeking, which had caused delays or led to not seeking care at formal facilities. The causes related to expectations of high medical costs, perceived cause of illness and, linked to this, the perceived likelihood of recovery. The level of disagreement and the delays this created varied, with the most severe disagreement reported by a woman who attributed her husband’s lack of engagement in the treatment of their child to one of the reasons why she later divorced him. Disagreement between spouses was however less frequently reported than disagreements with in-laws and parents. Mothers described how they in pragmatic ways coped with different preferences in the household:

When my parents prefer to do a traditional treatment I will not intervene. To avoid quarreling I agree with them first. If the treatment does not work they accept and let me go to consult with a doctor [ID 3].

Another young mother exemplifies her economic dependency on relatives when seeking care for her child:

"Everything depends on the decisions made by my parents because they are responsible for all expenses. I never disagree with my mother. During the illness of my son they agreed with me to take my child to the hospital; my husband’s parents however did not [ID 2]."

Both men and women stressed the importance of letting their parents influence the path of treatment, due to their experience and responsibility within the household. In some households the praying to household spirits could be carried out in parallel with leaving for the hospital and did thus not imply any delay while other households insisted on completing spiritual obligations before taking the child out of the village.

Secondly, a source of concern for all households was costs, which can be broken down into a lack of access to cash and a lack of mobilization of household and community resources. In seeking formal treatment, one of the first comments respondents gave in interviews was the concern over cash and the trade-offs between selling off assets, taking informal loans or seeking cheaper providers. While all providers charge for treatment, spiritual healing and informal treatment were associated with greater economic flexibility in terms of credit, payment in kind and for some herbalists a small consultation fee is requested while a larger sum is only requested upon recovery. Formal healthcare is known to require up-front payments as a condition for treatment. A husband arriving at the referral hospital with his wife, who was facing complications during labor, describes:

"When we arrived at the hospital, I gave a letter from the district hospital to the doctor there. The doctor did not ask me any questions; he took my wife to the delivery room. Then he came to me and said that my wife needed a C-section immediately and asked if I had money as she also needed blood transfusion during the surgery. It might be about 2 million kip [approximately $US 250]. I told the doctor to go ahead with the C-section so my wife would survive [ID 7]."

The uncertainty regarding the final cost of treatment was a particular concern, including formal and informal payments during admission, resulting in some patients leaving the hospital when resources were exhausted rather than when treatment was completed.

Third, health beliefs and in particular the matching of known or unknown symptoms to causes of illness emerged as another factor in selecting provider. Certain symptoms such as seizures were associated with spiritual causes and implicated healers. But more importantly, the transition from a perceived “normal” to a “severe” fever was difficult for many caregivers to diagnose and it was in this part of the interview that parents often were critical to their assessment of how sick the child had been. One mother and aunt describes the loss of her two year old nephew whom she took care of:

"The cause of his death was hot fever. By the first day I bought some liquid from a local pharmacy [liquid paracetamol] for him. He took the liquid three times a day. By the first and the second day he was better but by the third day he had a severely high fever and heavy breathing. I gave him the same liquid but it did not help anymore and he got worse and worse. I was supposed to meet a village health worker in the afternoon but it was too late. He passed away. I thought he had a normal fever and therefore I did not take him to the village health volunteer and bought some medicine at the local pharmacy instead [ID 1]."

Finally, caregivers’ uncertainty of social norms in the health facilities was a frequently mentioned barrier to seeking formal providers. The discomfort in talking to a doctor did not only refer to the lack of a common language for Non-Lao speakers but also to illiterates’ embarrassment about their inability to read the signs indicating where to go in the facility and their unfamiliarity with the expectations regarding caregiver behavior during consultations and admissions. One woman described how it was only during her third prenatal consultation that she finally told the nurse about the pain that worried her and had made her seek care in the first place.

### Delay in reaching care

Given the oversampling of communities with good access to health facilities, delays in reaching health facilities were more often mentioned than anticipated. The normal time to walk to a facility increases substantially when carrying a child. One mother says:

"I could not take her [young daughter] to the hospital because it is very far from the village and we did not have a vehicle. It would take one day to walk [ID 14]."

In analyzing how these delays came about, it became clear that the physical barriers to access were not the only processes that affected a household’s coping with distance. It went hand in hand with a concern about leaving the village. Fear of dying outside the community was pervasive along with concerns that the patient was too weak to be transported. Both among Buddhists and animist groups, the additional vulnerability associated with dying outside the village involved a risk of misfortune for the family, requiring mitigating spiritual ceremonies in addition to the logistical challenges in transporting the deceased back to rural communities. Caregivers thus highlighted the difficult trade-offs between wishing to seek care and assessing that the sicker the patient became, the higher the risk of dying on the way.

### Delay in receiving care

Caregivers who had experienced delay in receiving care mentioned very different issues that directly or indirectly reflected delay. Health worker absenteeism at health centers upon arrival, a reported low capacity of health workers to treat emergency cases, and lack of medical obstetric emergency equipment posed extreme challenges according to caregivers. Coping with the lack of support from referral services emerged as a frequent cause of delay to receiving care among the caregivers who had experienced an emergency situation at a health facility. Two of the children who, during the first five months of life were admitted to a district hospital several times but were not cured, were, during their last weeks of life, recommended to seek care elsewhere. The parents expressed regret for not realizing earlier that the district hospital could not treat their children. A husband describes the actions leading up to the death of his first wife and 7th child:

"Prior to the death of my wife, she had been pregnant for 8 months, she was still working all day transplanting rice; she would come home afterwards. After dinner, we went to sleep as usual, and she felt no pain at all. At 3:00 am she had a bleeding. She got up and was bleeding heavily. Then I carried her to the health center, but the staff there said that her situation was too serious for them to treat and they suggested that I take her to the hospital. I spent about 30 minutes to arrange for a vehicle and to inform relatives before taking her to the hospital. We took her on a small tractor belonging to relatives and travelled to the district hospital. We arrived at the hospital at about 7:00 am."

"As soon as we arrived, the health staff and the doctor at the hospital came out to see her. Then they took her to the delivery room, gave her intravenous injection drip, and injections to prevent bleeding. The health staff checked her cervix and checked the unborn baby several times. They said that all blood had come out, but the baby didn’t. At that time it was only me, my wife and her younger brother who stayed at the hospital. The other relatives had already gone back to the village."

"From 7:00 am to 3:00 pm, three or four health staff took turns in watching my wife. They checked the baby, gave injections to force the baby to come out, and gave her three bags of intravenous injection drips and oxygen mask. At the very end my wife was very exhausted because of the heavy bleeding. Then the health staff said that they could not provide her further treatment, and suggested that I took her to the provincial hospital or some other health facility that had surgery equipment available and could give blood transfusion. At that time my wife was still able to speak. I therefore left to go out to rent a vehicle at the bus station. I spent about 30 minutes to arrange for the vehicle and I left my wife alone at the hospital. As soon as I came back to carry her to the vehicle, she passed away……If the health staff at the district hospital had told me in the morning that my wife was extremely seriously sick and had suggested that I took her directly to the other hospital, she might not have died [ID 6]."

One woman describes her experience of not being referred to another hospital during her delivery of twins:

"My relatives were afraid that I would die, and so, they were upset with the doctors twice and asked to see other doctors at another hospital. But they did not let me go because they believed they could carry out my delivery safely……The doctor told my husband that we had to take our chances and that the babies and I would be safe if there was any justice [ID 12]."

The twins survived the delivery but passed away during the two first months of life in the home. For most caregivers it was not perceived as a viable option to complain directly to the health staff. Leaving the facility prematurely was an easier alternative to opt for which contributed to children dying on the way home from the hospital.

## Discussion

In this study it was found that many caregivers, having lived through the loss of a child or spouse, experienced many constraints during the fatal illness episodes. All three delays were found to have influenced the death of the mothers and children even though the first delay was most frequently identified. To our knowledge, three other studies have applied the three-delay framework in child mortality investigations. Two of these analyzed specifically infants: newborn deaths [40] or hospital perinatal deaths of infants weighing less than 2000 grams [39] and a third one considered under-five year olds [26]. In comparison, the two studies on infants found fewer decision-related delays and higher proportions of third delays than in this study. This discrepancy is likely to be due to differences in target groups, in the higher number of caregivers seeking care at the hospital or other contextual differences between Laos and the East African countries in which these studies were conducted. Our results are consistent with the study on under-five year olds in terms of the caregiver constraints in recognizing severe symptoms that contributed significantly to the delay in the decision of actually seeking care.

Our open-ended approach allowed us to identify very different types and degrees of influences on the processes during all the stages of healthcare-seeking. Further, each step in the healthcare-seeking process was ascribed specific social meanings by the caregivers interviewed, and some of those related to a very broad spectrum of social aspects of which some touched upon deeply rooted perceptions of how parents and relatives are expected to behave during a health crisis. The preferences for local treatment of sick children was, for example, caused not only by expectations of lower medical costs but also as a mitigating strategy to avoid inflicting harm on other family members if the child was to die outside the community without a set of spiritual requirements complied to. Illness and treatment decisions thus confirmed social roles and relationships in everyday lives [45]. The decision to seek formal care was made in the hope of recovery and the hospital medicines and procedures objectified this hope. Studies have documented the social connotations of biomedicines as globally increasingly powerful [8,46,47].

Further, the interdependency between access to cash and intra-household decision-making processes emerged as an important constraint in health-seeking behaviors. When care became a question of spending a potentially large amount of the household savings or assets, mothers were less likely to control the path of treatment. Women’s autonomy within the household has been identified as a determinant for maternal mortality [48] and the findings of this study support this notion – that household consensus cannot be assumed [49,50]. Mothers’ social space to influence the therapeutic paths of children has to be specifically analyzed. However, the decision-making processes are not only related to gender but also generation. The social organization and roles ascribed to mothers-in-law in a patrilineal descent system meant that elder women were not automatically sources of support during the difficult situations of very sick children and costly options of care. The group of people influencing the decisions are referred to in the anthropological literature as the “therapy management group” [51] which illustrates the social character of healthcare decision-making. From a health planning perspective it implies that contextual gender- and generational-related - rather than only individual - aspects of decision-making during disease are significant [9].

In addition, the recognition of symptoms as severe was identified by caregivers as a very difficult task. The transition from what was experienced as frequent and therefore “normal” fevers of young children to life-threatening fevers was recalled as a very difficult process. Severity of symptoms has in several studies been found to be a challenge for caregivers [26,41,52].

The consultation at a healthcare facility was framed by uncertainty related to recovery, medical costs as well as expectations regarding how health staff would respond. The social framework associated with healing by traditional providers was to some degree extended to the formal facilities and uncertainty was mitigated by family members accompanying the patient. Caregivers often spontaneously mentioned who had been with them in the different stages of the episode and being alone with the patient at a hospital was clearly defined as a vulnerable situation. It has been found that “knowing a doctor” and thus having some sense of what is expected can impact not only on healthcare-seeking behavior but also the likelihood of recovery [53]. This reinforces the argument that tacit knowledge of the caregiver behavior required in a medical facility is an important factor to look into for future interventions [7,54,55]. The implication of this finding is, given the ethnic and cultural diversity in Lao PDR, that the healthcare system is faced with a huge task in accommodating this cultural diversity [56]. The limited availability in remote areas of nurses and doctors speaking one or more of the ethnic minority languages is possibly one factor contributing to the perceived differences between patient and doctor. The Ministry of Health of Lao PDR is currently addressing this gap through purposive recruitment of students from remote areas but the program is challenged by the low education levels of the applicants when recruited.

There is an additional important implication of the findings, which relates to financing of the health system. Economic barriers for seeking formal healthcare were identified as one of the major reasons for delay in seeking care or not seeking care at all. A study in Laos found that health shocks impose a greater damage on the well-being of poor households than among the better-off households [57]. This is not surprising as it has been estimated that 67 percent of all health expenditures are financed through out-of-pocket payment in Laos [58]. Removal of direct payments for care has shown encouraging results on health-seeking behavior in other low-income countries even though it does not prevent all delays [59]. A program to this effect is currently being developed in Laos, although there are several challenges relating to financing and implementation that need to be addressed [60].

### Implications for future research

The need for innovative approaches in creating reliable and useful data on reasons for child and maternal death has been acknowledged in the development of methodologies like the verbal autopsy and more recently the social autopsy. The verbal autopsy is a method for establishing the probable medical cause of death through structured interviews with caregivers in settings where civil registration systems are absent [3,61,62]. The social autopsy complements this analysis by identifying social, behavioral and health system determinants of deaths, also through structured interview processes [21,26]. While new standards of verbal autopsy have been established, standardization of the social autopsy is ongoing [62]. This having been said, even in the case of verbal autopsy, the need for increased flexibility when gathering data is emphasized by a recent study on maternal deaths [63]. That study also showed that when comparing the responses provided by caregivers in answering open-ended and closed survey-inspired questions, the former identified contributing factors to death in up to 25 percent more of the caregiver interviews than the latter. This was the case, despite the fact that the range of social and cultural factors that were probed was restricted. The challenges in applying closed questionnaire-based methods to capture fatal illness episodes were also identified in a study on the spirit child phenomenon in Ghana [16]. There it was recommended that verbal autopsy interviewers should pay particular attention to local death cause classifications, pre-and post-mortem. It was found that perceived causes of death changed fundamentally during the illness episode and the authors therefore recommended that uncertainty should trigger additional interview questions.

The use of open-ended questions and of probing in our study allowed the caregiver to raise very broad factors that were relevant to them for the loss of a child or a mother/spouse. This finding stresses that health-care seeking is a complex process and underscores the importance of broadening the field of inquiry. In order to enhance the comprehensiveness of the analysis on maternal and child deaths, aspects that capture the gender and poverty related factors, that are recognized to be major social determinants of morbidity and mortality [64], could be included. The inclusion of an “open-ended illness narrative module” in sub-samples, where broad social and cultural contributory factors are studied, could potentially add value as a complement to the structured verbal and social autopsies.

The development and integration of such a tool would meet the challenges including that of finding in-country interviewers with not only the skills needed to perform open-ended interviews but also those needed to handle the sensitive issues of maternal and child loss. Additionally, skilled in-country analysts would be needed to ensure that the richness of the data is not lost or misunderstood. All these challenges are currently overcome in verbal and social autopsies, or their impact at least controlled, and could most likely be managed in this approach as well.

In deciding the timing of interviews with caregivers who have lost a child or wife, the sensitive nature of the interview has to be considered. Caregivers’ emotional difficulties in narrating the illness episode four to six weeks after the death was noted in one study in Uganda [55]. Recall error could be a concern in open-ended illness narratives but the time span is probably relatively broad. In one study it was found that recall was unaffected within 21 months [24]. During data analysis in this study it was not possible to discern any major systematic differences between the losses of children or mothers less or more than 24 months ago. The stages of bereavement that families go through after maternal or child losses affect their understanding of what happened and why it happened, which in stages of blame could result in increased reporting of delays in receiving care at the health facility. These are biases that would be very difficult to control but would speak for a longer period between the fatal illness and the interview [21]. A longer recall period gives the caregivers the opportunity to create some distance to the loss [25] and with that provides a better prospect to elaborate the rationales for health-care seeking prior to death.

### Study limitations

The relative frequency of any type of delay identified is uncertain and cannot easily be compared or extrapolated to other settings. But this does not affect the finding that a large number of delays were identified with the narrative approach [63]. The three-delay framework could be modified due to its inbuilt assumption that “no delay” in deciding to seek care would imply survival of the child or mother. This was clearly not the case in this study where delays in reaching and receiving care also were crucial. The ethnic mosaic in the study districts makes sampling a challenge and we cannot claim to have reached saturation in terms of ethnic diversity. The limited number of ethnic groups included in this study indicate the importance of taking on this challenge in the provision of service delivery [56].

## Conclusions

Maternal and child deaths in poor rural districts in Lao PDR occur in the context of highly complex social dynamics within households, with added complexity from the interaction with health care staff. The application of the qualitative open-ended illness narrative interview enabled the identification of important constraints in health seeking that possibly contributed to the fatal health outcome. It was revealed that decision-making delays were often influenced by caregivers’ concerns of maintaining good family relations, of mitigating the economic consequences of seeking care, and of reducing the risk of getting low quality treatment when selecting healthcare provider. Additionally, the medical and normative responses from health providers during illness episodes affect caregivers’ decision to continue to seek care. Delays in reaching a health facility with a sick child or spouse affected also households with good access to health facilities due to the limited transport available in rural areas. Delays in receiving care were especially difficult when referral to another facility was required and this reveals the constraints in the current health system. The identified delays reflect the socio-cultural priorities of the caregivers but also a health system that faces challenges in providing services that are trusted and located close to the communities. The experiences of the caregivers illustrate how the social processes are at play not only in the community and within the households but, equally importantly, in the interactions with health workers.

The open-ended applied anthropological approach in interviews with caregivers identified health care delays in the majority of fatal episodes and broadened the scope of the field normally considered relevant for understanding such delays. Caregiver experiences on fatal illness give us insights into the complexity and socio-cultural context surrounding maternal and child mortality, which are useful in future development and interpretation of mortality data.

## Endnotes

^a^The names of the districts are kept anonymous since there is only one district hospital in each district and we are presenting data on delays in received care.

^b^All names of health facilities have been replaced. They are referred to as levels in the health system as follows: health center, district hospital, provincial hospital, national hospital and hospital in Thailand.

## Competing interests

The authors declare that they have no competing interests.

## Authors’ contribution

HMA and ML conceived and designed the study. HMA and BK collected, analyzed and interpreted the data. All authors made contributions to the manuscript and have read and approved the final manuscript.

## Authors’ information

HMA was funded by the World Bank during data collection and by Karolinska Institutet when working on this study. ML is funded by the World Bank. BK is funded by Indochina Research. LL is funded by Karolinska Institutet.

## Pre-publication history

The pre-publication history for this paper can be accessed here:

http://www.biomedcentral.com/1472-6963/12/477/prepub
